# Supplementary material for: The Use of Psychotropic Medication in Pediatric Oncology for Acute Psychological and Psychiatric Problems: Balancing Risks and Benefits
Source: Children (Basel). 2022 Nov 30;9(12):1878. doi: 10.3390/children9121878 (PMC9777172; doi:10.3390/children9121878)
Supplement: Supplementary file 1 [file children-09-01878-s001.zip › children-1995940-supplementary.pdf]

Supplementary material Children 1995940

A questionnaire was sent out to Italian pediatric oncology centers

The questionnaire took about 10 minutes to be completed and was created and agreed upon in the first round of consultation by the workgroup. The questionnaire aimed to obtain global information on the use of several types of psychotropic drugs in the pediatric oncology setting. The medications listed on the questionnaire included: selective serotonin reuptake inhibitors (SSRIs), fluoxetine, paroxetine, sertraline, citalopram, and escitalopram; the dual antidepressant, venlafaxine; typical antipsychotics, haloperidol, and chlorpromazine; the atypical antipsychotic drug risperidone; anxiolytics (for simplicity, no specific drugs were listed as there are many different types).

A total of 28 pediatric oncologists and 12 child mental health specialists belonging to 16 centers responded to the survey. The questionnaire was anonymous, and participants could answer online or in paper format; 40% completed the survey online and 60% responded in paper format.

The questionnaire used in support of the workgroup

.

Question	Response options
**1. Are psychotropic medications prescribed in your unit**	Yes; No; Rarely
**2. How often do you prescribe psychotropic drugs?**	Never; Sometimes; Often; Always
**3. Do you consult with a child psychiatrist, psychologist, or other specialists capable to evaluate the mental health needs of the patient?**	Never; Not often; Often; Always
**4. If yes, whom do you consult?**	Yes; No; If yes, who?
**5. Which medications do you typically prescribe?**	Fluoxetine; Sertraline; Paroxetine; Citalopram; Escitalopram; Venlafaxine; Risperidone; Olanzapine; Haloperidol; Chlorpromazine; Anxiolytics; Benzodiazepines; Other………………………..
**6. How do you establish what medication to use?**	
**7. What signs and symptoms do you use when you decide to prescribe) (circle all that apply)**	General sadness; Major Depression; Anxiety; Prior history of psychopathology; Behavioral dissociation, Psychosis
**8. Is age a factor in prescribing specific medications? For which age group do you prescribe**	If Yes; < 10; 10-14; 15-18; > 18; Independent of age; As needed
**9. When during treatment are you most likely to prescribe?**	During the first 6 months; After the first six months up to the first year; After the first year
**10. Which specific symptoms lead you to a diagnosis of depression?**	
**11. Which specific symptoms lead you to a diagnosis of psychosis?**	
**12. Are patients being monitored during treatment?**	Never; Sometimes; Often; Always
**13. What do signs and symptoms you monitor and with what frequency?**	
**14. Are prescribing practices of psychotropic medications influenced by a lack of specific knowledge concerning their use in children and adolescents?**	Yes; No; If yes, how?
**15. What additional resources would be useful to better manage the use of psychotropic medication in pediatric cancer patients?**	

Analysis of the questionnaires

All responses were entered into a database with text fields for open-ended questions. Categorical variables were analyzed with descriptive statistics. Free text comments and answers to open-ended questions were coded, organized into broad categories, and examined qualitatively. SPSS version 25 was used to analyze the data. After careful analysis of the literature and existing specific and more general guidelines, together with analysis of the data collected from the questionnaires, experts entered a series of four cycles of careful consideration of patient, treatment, and disease-related variables resulting in specific clinical questions.

1. Are psychotropic drugs prescribed and with what frequency

   Almost all participants declared the prescription of these medications, while only a tiny portion (8%) answered that psychotropic medication was prescribed rarely or not at all (**Fig.1A**). Even though most centers reported the use of psychotropic medication for their patients, to the question at *what frequency are psychotropic drugs prescribed?*', only 9% reported a frequent use (**Fig.1B**).

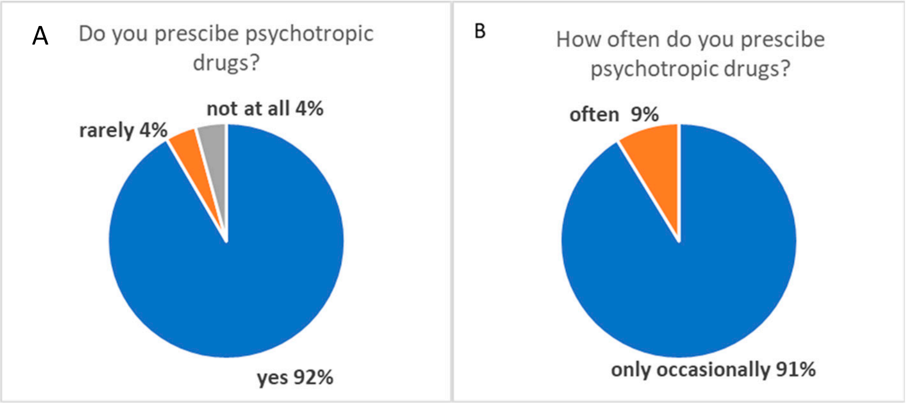

Figure 1

2. Drugs usually prescribed

Serotonergic antidepressants were prescribed by 41% of the participants followed by anxiolytic drugs (34%) and antipsychotic drugs (23%) (**Fig. 2A**). Fluoxetine and the newer SSRI Citalopram were prescribed most, followed by Sertraline and Paroxetine (**Fig. 2B**). Tricyclic drugs were used sparsely (2%). Concerning antipsychotic drugs, of the 23% prescribing them, 84% declared to prescribe haloperidol or chlorpromazine (8%), both belonging to the class of typical antipsychotic drugs, while only 8% recurred to the atypical antipsychotic risperidone.

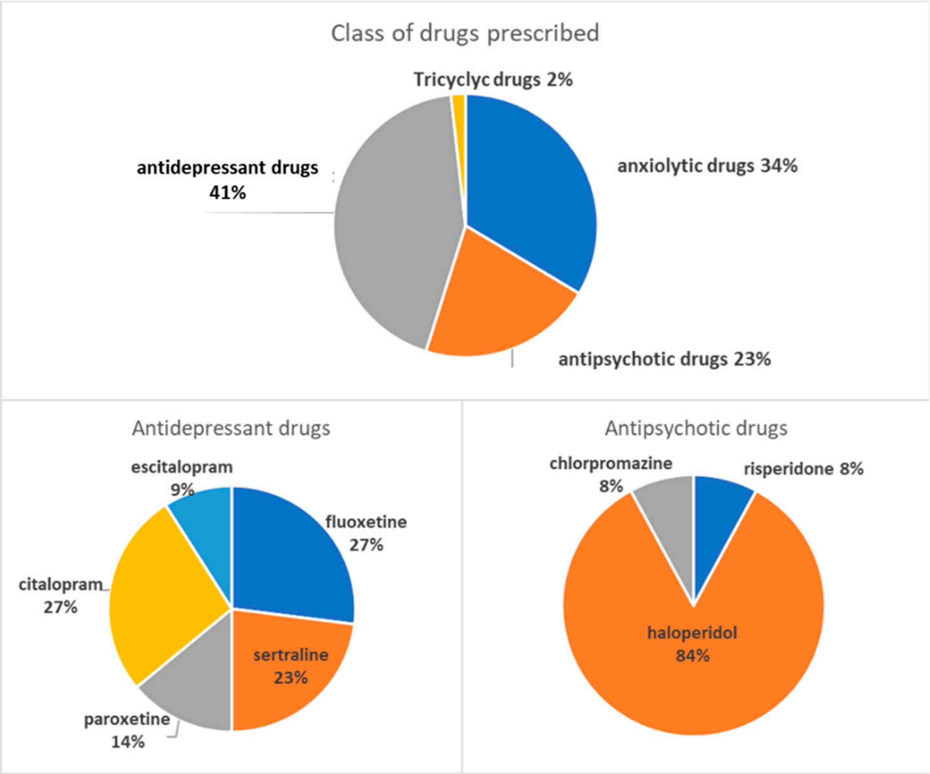

Figure 2

3. Signs and symptoms used to decide what to prescribe

The signs and symptoms most often used to prescribe psychotropic drugs were manifestations of general sadness (9%), depression (20%), anxiety (24%, pre-existing psychopathology (17%), dissociative behavior (17%), and psychosis or delirium (13%) (**Fig. 3**)

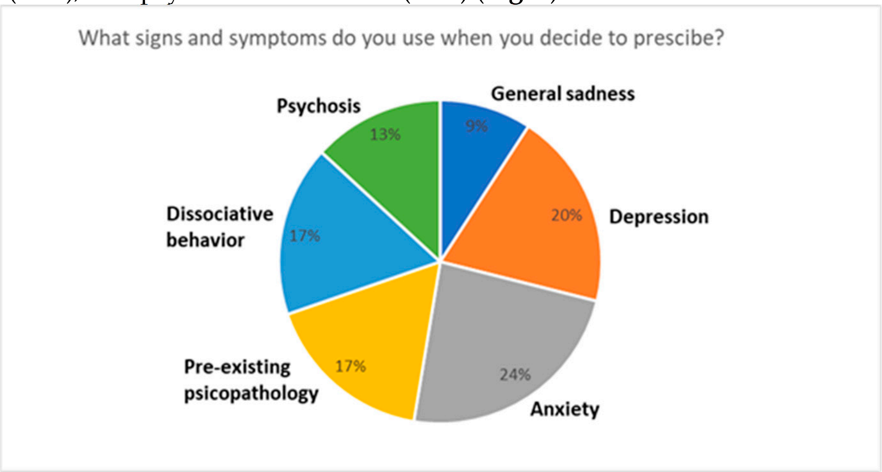

Figure 3

4. Age as a factor in prescribing psychotropic medications

Twenty percent of the respondents declared to prescribe psychotropic drugs independent of the age of their young patients. psychotropic medications were primarily prescribed to patients between 14 and 18 years of age (21%) and early adolescent patients (13%).

5. Treatment phase in which drugs are mainly prescribed

Almost 50% (48,4%) of the respondents declared to prescribe psychotropic medications during the first six months of treatment to treat the behavioral side effects caused by corticosteroids. Much less use was made during the following six months (8%) or beyond the first year of treatment (18%).

6. Consultation with a psychiatrist, psychologist, or other specialists to evaluate the mental health needs of the patient

Most respondents always (67%) or mostly (21%) consulted with a mental health specialist before prescribing psychotropic drugs. However, more than 10% of onco-pediatricians rarely seek advice, and 3% do not consult with mental health professionals before prescribing psychotropic drugs. Mental health consultation mostly involved child psychiatrists (44%) or psychologists (38%). In 15% of the cases, adult mental health professionals, psychiatrists (9%), and neurologists (6%) were asked for a consultation.

7. Symptoms used to diagnose depression

The symptoms that mainly directed the respondents to a diagnosis of depression included sleep disturbances, severe insomnia, lack of appetite, and eating problems. Other symptoms observed frequently were apathy and refusal of relationships, especially the unwillingness of peers. Extreme symptoms, such as an intense and sudden drop in mood or recurrent suicidal thoughts, were reported to be less present during the first six months. However, when present, these prompted an instant use of antidepressant drugs.

8. Symptoms used to diagnose psychosis

Symptoms of aggression, extreme hyperactivity, irritability, and dissociative behavior were the main symptoms that led them to a diagnosis of psychosis.

9. Signs and symptoms used to monitor the efficacy and side effects of the treatments

Most centers monitored for the clinical efficacy of psychotropic medication in reducing behavioral symptoms, often using standardized behavioral tests and scales. Emphasis was also placed on monitoring drug metabolism and interactions with other treatment-related drugs (i.e., chemotherapy) and psychotropic drug-induced side effects. Another problem reported as important, especially in adolescent patients, was the necessity to monitor compliance. Furthermore, some respondents indicated that they felt it difficult to evaluate and separate clear psychiatric problems from emotional reactions related to the child or adolescent’s disease treatment regimens.

10. Additional resources suggested managing better the use of psychotropic medication in pediatric cancer patients

Many respondents pointed to a general lack of specialists present or on-call competent in managing the severe acute behavioral and emotional emergencies of their patients. Almost all centers called for an enhanced presence of mental health specialists capable of in-depth psycho-diagnostic assessment and advice on how to treat children and adolescents with SABEPs. One solution suggested by many centers is the development of "various treatment strategies", which would allow the pediatric oncologist to deal adequately with the emergency in the presence of an acute and critical problem. Furthermore, many participants asked for additional training in how to deal with age-specific manifestations of treatment or tumor-related behavioral and emotional problems. The respondents also suggested increasing the number of experts, enhancing mental health problems-related knowledge, and providing practical advice to deal with critical and acute situations without specialized personnel.
